# Linkage disequilibrium blocks, haplotype structure, and htSNPs of human CYP7A1 gene

**DOI:** 10.1186/1471-2156-7-29

**Published:** 2006-05-18

**Authors:** Kaori Nakamoto, Shuang Wang, Robert D Jenison, Grace L Guo, Curtis D Klaassen, Yu-Jui Yvonne Wan, Xiao-bo Zhong

**Affiliations:** 1 Department of Pharmacology, Toxicology, and Therapeutics, University of Kansas Medical Center, 3901 Rainbow Boulevard, Kansas City, Kansas 66160, USA; 2 Department of Biostatistics, Mailman School of Public Health, Columbia University, New York, NY 10032, USA; 3 Inverness Medical-Biostar, Louisville, CO 80027, USA

## Abstract

**Background:**

Cholesterol 7-alpha-hydroxylase (CYP7A1) is the rate limiting enzyme for converting cholesterol into bile acids. Genetic variations in the CYP7A1 gene have been associated with metabolic disorders of cholesterol and bile acids, including hypercholesterolemia, hypertriglyceridemia, arteriosclerosis, and gallstone disease. Current genetic studies are focused mainly on analysis of a single nucleotide polymorphism (SNP) at A-278C in the promoter region of the CYP7A1 gene. Here we report a genetic approach for an extensive analysis on linkage disequilibrium (LD) blocks and haplotype structures of the entire CYP7A1 gene and its surrounding sequences in Africans, Caucasians, Asians, Mexican-Americans, and African-Americans.

**Result:**

The LD patterns and haplotype blocks of CYP7A1 gene were defined in Africans, Caucasians, and Asians using genotyping data downloaded from the HapMap database to select a set of haplotype-tagging SNPs (htSNP). A low cost, microarray-based platform on thin-film biosensor chips was then developed for high-throughput genotyping to study transferability of the HapMap htSNPs to Mexican-American and African-American populations. Comparative LD patterns and haplotype block structure was defined across all test populations.

**Conclusion:**

A constant genetic structure in CYP7A1 gene and its surrounding sequences was found that may lead to a better design for association studies of genetic variations in CYP7A1 gene with cholesterol and bile acid metabolism.

## Background

Cholesterol 7-alpha-hydroxylase (CYP7A1) catalyzes the first reaction in the cholesterol catabolic pathway in liver. This pathway converts cholesterol to bile acids, which is the primary mechanism for the removal of cholesterol from the body. The CYP7A1 catalytic reaction is the rate-limiting step and the major site for regulating homeostasis of cholesterol and bile acids. The gene encoding CYP7A1 was cloned by using a rat homolog probe [1] and mapped to chromosome 8q11 [2]. The CYP7A1 gene spans about 10 kb and contains 6 exons, 5 introns, one 5'-UTR, and one 3'-UTR. In its 5' flanking region, consensus recognition sequences for a number of transcription factors were identified [2]. A TATA box and a modified CAAT box were also identified in the promoter region of the CYP7A1 gene [3]. Numerous laboratories have illustrated a multiplex nuclear receptor mediated network that controls CYP7A1 gene expression and maintains cholesterol and bile acid balance [4]. Within this network, nuclear receptors of farnesoid X receptor (FXR), liver X receptor (LXR), retinoid X receptor (RXR), small heterodimer partner (SHP), and liver receptor homologue 1 (LRH1) are involved in a positive-versus-negative regulation. Using a FXR-deficient (-/-) mouse model, we have demonstrated feedback suppression on CYP7A1 gene transcription by FXR [5,6].

Genetic variations in the CYP7A1 gene associated to disorders of cholesterol and bile acid metabolism have been studied extensively in different laboratories. Most studies have focused on a single nucleotide polymorphism (SNP) in the promoter region of the CYP7A1 gene. This is an A/C transversion polymorphism at -278 from the translation initiation codon, or -204 from the transcriptional start site. This polymorphism was first reported by Wang et al. [7] to link to high plasma low-density lipoprotein cholesterol concentrations. Association of this polymorphism to plasma lipid levels, hypertriglyceridemia, hypercholesterolemia, and risk to arteriosclerosis, gallstone disease, and colorectal cancer has been studied in adults and children in Caucasian and Asian populations with conflicting results [8-19]. A CYP7A1 enzyme deficiency caused by a homozygous 1302–1303 delTT deletion mutation in CYP7A1 exon 6, leading to a frameshift (L413fsX414), has been linked to a hypercholesterolaemic phenotype [20]. The information has indicated that genetic variations in the CYP7A1 gene have high impact on human cholesterol metabolic regulation and human health; however, these studies have mainly focused on a single polymorphism or a mutation. Linkage of genes for a complex disease relies on having *a priori *knowledge of linkage disequilibrium (LD) blocks and haplotype structure to identify polymorphisms that are associated with the disease. Therefore, it is important to determine whether there are LD blocks existing in the CYP7A1 gene in different populations. This information can be used to identify a set of haplotype-tagging SNP (htSNP) markers that can be used in an association study.

The LD blocks and haplotype structure of CYP7A1 gene can be firstly defined in three general human populations of Africans, Asians, and Caucasians using a public-available database generated by the International HapMap Project [21]. The HapMap LD patterns and haplotype structure can serve as reference to select htSNPs for an association study. LD patterns and htSNPs defined by the HapMap Project are transferable to other populations in some loci, but may vary significantly in other loci [22]. To test whether the htSNPs identified in the HapMap populations are useful for association studies in other populations, we analyzed LD patterns and haplotype structures of CYP7A1 gene in both Mexican-American and African-American populations using the selected HapMap htSNPs. Mexican-American is the fastest growing population in USA, but genetic study on this population is extremely limited. Mexican-American genetic background is a mixture of European American (50–60%) (mainly Spanish), American Indian (30–40%), and African (<5%) [23]. African-American is the major minority population in USA and has an admixture genetic background from African and European Americans [24]. Genotyping of the selected htSNPs on these two populations can provide verification of transferability of the HapMap htSNPs among populations.

## Results and discussion

### Linkage disequilibrium blocks and haplotype structures of CYP7A1 gene in Caucasians, Africans, and Asians

**Figure 1:**
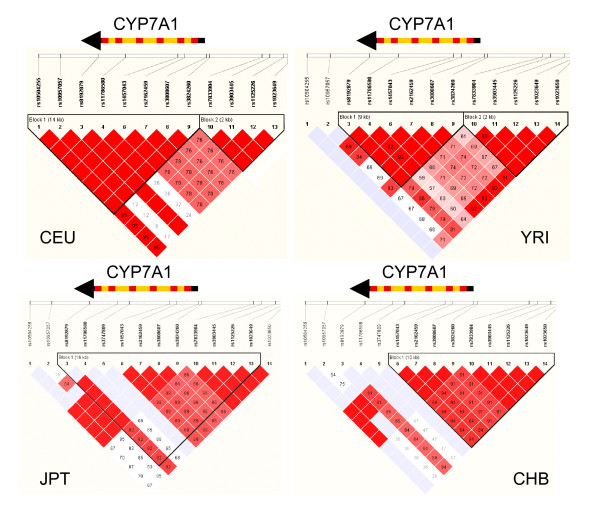
Linkage disequilibrium of the SNP markers in the CYP7A1 gene in the HapMap populations of CEU, CHB, JPT and YRI. A standard color scheme is used to display LD with bright red color for very strong LD (LOD = 2 D' = 1), white color for no LD (LOD<2, D'<1), pink red (LOD = 2 D'<1), and blue (LOD<2 D' = 1) for intermediate LD.

**Figure 2:**
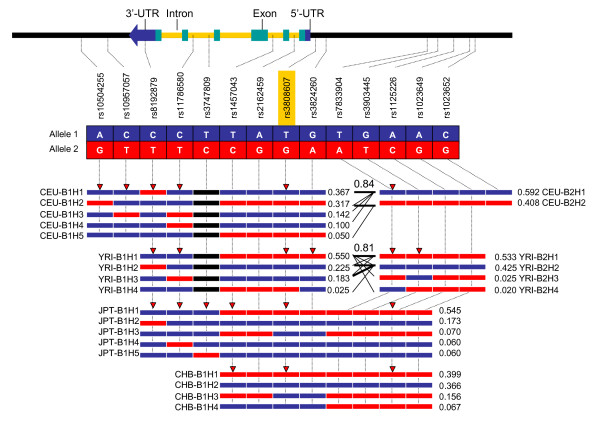
Haplotype frequencies of the HapMap selected SNPs in the CYP7A1 gene in CEU, YRI, JPT, and CHB. In each haplotype, blue bars represent allele 1, whereas red bars represent allele 2 for correlated SNPs. Black bars indicate that the SNPs are not present in this population. Numbers next to each haplotype bar are haplotype frequencies. Up-side-down red triangles indicate htSNPs in the populations. In the crossing areas, a value of multiallelic D' is shown to represent the level of recombination between the two blocks.

A LD block is found in the HapMap Caucasians (CEU) spanning a 14-kb region from the proximal promoter (rs3824260) to the 3'-downstream (rs10504255) of the CYP7A1 gene (Figure 1. CEU-B1). A similar LD block from rs3824260 to the 3'-downstream was also reported in a Swedish population [18]. About 4.4 kb upstream from rs3824260, there is another LD block (CEU-B2) crossing a 3-kb region at the distal promoter region. Recombination between the two blocks is 0.84. Only five haplotypes with a frequency > 2% exist in CEU-B1 (Figure 2. CEU-B1H1 to CEU-B1H5). CEU-B1H1 and CEU-B1H2 are two common haplotypes, together representing a total of 68% of the haplotype frequency in CEU-B1. CEU-B1H1 carries common alleles at all markers except rs8192879 in 3'-UTR, whereas CEU-B1H2 is composed of less common alleles at 5 out of 8 loci. In CEU-B2, there are only two types of haplotypes (CEU-B2H1 and CEU-B2H2). CEU-B2H1 carries common alleles at all SNP loci, whereas CEU-B2H2 has less common alleles. A similar LD pattern is found in the HapMap African YRI (Figure 1), but the larger LD block (YRI-B1) is slight shorter (9 kb from rs8192879 to rs3824260) than CEU-B1. The haplotype structure is also similar between YRI and CEU, however, the frequency of each haplotype is different. The most common haplotype (55%) in YRI-B1, YRI-B1H1, has identical haplotype structure with CEU-B1H2, whereas the second common haplotype in YRI-B1, YRI-B1H2 (22.5%), is the same as CEU-B1H1. YRI-B1H1 and YRIB1H2 together add up to 77.5% of the total haplotypes in YRI-B1. In YRI-B2, the dominant haplotype YRI-B2H1 has the same haplotype structure as CEU-B2H2, whereas less common haplotype YRI-B2H2 is identical to the common haplotype CEU-B2H1. A similar recombination (0.81) is also found between YRI-B1 and YRI-B2. In CHB and JPT, only one LD block is found from the distal promoter to a part of the CYP7A1 gene. Although the JPT-B1 (16 kb, from rs8192879 to rs1023649) is larger than CHB-B1 (10 kb, from rs1457043 to rs1023650), LD is weak between rs8192879 in intron 4 and rs1457043 in intron 2 in JPT. CHB and JPT share almost the same haplotype structure within the block. JPT-B1H1, JPT-B1H2 and JPT-B1H3 are the same as CHB-B1H1, CHB-B1H2 and CHB-B1H3, respectively. Only CHB-B1H4 (6%) is unique in CHB.

In comparison of LD and haplotype structure among the HapMap populations, strong LD is found from the distal promoter region to intron 2 of the CYP7A1 gene across the HapMap populations. Two common haplotypes with complete opposite alleles at all loci (common-versus-less common alleles) within this region count for more than 85% of total haplotype frequencies in all four HapMap populations. A diverted LD degree exists between intron 2 and the 3'-downstream region from high to low across CEU, YRI, JPT, and CHB.

### Genotyping of htSNPs in Mexican-Americans and African-Americans

Because of the strong LD in the CYP7A1 genes, some markers correlate 100% with each other in a population. Only a subset of representative SNPs is necessary for defining a haplotype. These SNPs can tag either neighboring markers or a set of common haplotypes within an LD block. The htSNPs in CYP7A1 were selected using Tagger, implemented in the HaploView 3.12, which combines the simplicity of pairwise methods with the potential efficiency of multimarker approaches [25]. The CYP7A1 htSNPs are different in the various populations (see upside-down red triangles in Figure 2 for each HapMap population). Some markers tag on all three populations, but others for only one or two. It has been suggested that the populations genotyped in the HapMap project may serve as reference populations for the selection of htSNP markers in association studies [26].

Nine SNP markers and one short deletion marker were selected (see detail in Table 3), in which, eight are htSNP markers defined by the HapMap populations, including rs3808607, a functional polymorphism at A-278C in the promoter region. Two functional mutations were also included. One is a two-base deletion in exon 6 (1302 delTT) causing a frame shift and CYP7A1 enzyme deficiency [20]. The other one is a C/T SNP in exon 3, causing an amino acid change at Asn233Ser. This is the only non-synonymous SNP reported in NCBI SNP database in the CYP7A1 gene.

To perform genotyping of the 10 markers in the Mexican-American and African-American populations, a high-throughput and inexpensive SNP genotyping platform was developed using thin-film biosensor chips. We have reported a microarray platform for genotyping both SNPs and microsattelite repeat on thin-film biosensor chips [27,28]. The thin-film biosensor chip has excellent sensitivity of detection and extremely low non-specific binding, making it an excellent platform for discrimination of polymorphisms [29]. A positive reaction (blue color signal) can be visualized over the unreacted background (gold color) by an unaided human eye, without any instrumentation. Once the chips are printed, they are robust. Several thousands of genotypes can be performed in a 96-well plate in a laboratory with a standard molecular genetics setting within a few hours. Cost for reagents and materials to genotype 10 CYP7A1 htSNPs, including genomic DNA isolation, PCR reaction, and SNP genotyping on the thin-film biosensor chips, is ~US$0.20 per SNP per sample. It is relative less expensive than other high-throughput genotype platforms, such as TaqMan or Real-time PCR.

**Figure 3:**
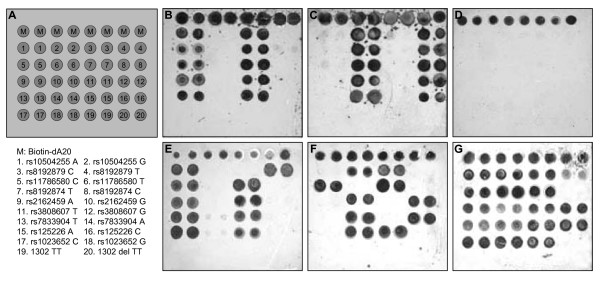
Genotyping of the 10 markers of CYP7A1 htSNPs and small deletion on thin-film biosensor chips. A. A design for arraying the capture probes on a thin-film biosensor chip. A pair of capture probes for each SNP were arrayed in duplicate next to each other with allele 1 left and allele 2 right. M indicates a positive control marker with 20 dATP and 3'-biotin. B. SNP discrimination with a pool of synthetic oligonucleotide targets of allele 1. C. SNP discrimination with a pool of synthetic oligonucleotide targets of allele 2. D. Negative control with no targets. E. A representative image showing genotypes of a Mexican-American individual with homozygous allele 1 for the most markers, but homozygous allele 2 for rs8192879. F. A representative image showing genotypes of a Mexican-American individual with homozygous allele 2 for the most markers, but homozygous allele 1 for rs18192879, rs11786580, rs8192874, 1302 TT. G. A representative image showing genotypes of a Mexican-American individual with heterozygous for the most markers except rs8192874, 1302 TT.

To verify genotyping specificity on the thin-film biosensor chips, a pool of the synthetic targets for allele 1 or allele 2 was applied to a chip for hybridization and ligation. After signals were developed, the result images were captured by a black-white camera on a Nucleosite™ Image Analyzer (Biostar, Inc., Louisville, Colorado). High specificity was achieved on these synthetic targets with unambiguous genotypes (see images in Figure 3B and 3C). A negative control showed the signals are target dependent (Figure 3D). As a positive control for genotyping, 12 HapMap DNA samples were purchased from Coriell Cell Repositories (Camden, NJ), which are one family trio from YRI (NA18500, NA18501 and NA18502); one family trio from CEU (NA06985, NA06991, and NA06993); three independent individuals from CHB (NA18524, NA18526, and NA18529); and three independent individuals from JPT (NA18940, NA18942, and NA18943). Genotypes of the 8 HapMap htSNPs in the 12 HapMap samples were determined on thin-film biosensor chips. A 100% concordance was obtained between the 96 genotypes generated by thin-film biosensor chips and the 96 genotypes downloaded from the HapMap database which are generated by Illumina Bead Assay.

To define the LD pattern and haplotype structures of CYP7A1 gene in Mexican-American and African-American populations, DNA samples from 90 healthy individuals for each population were randomly selected from our DNA bank. These DNA samples were collected by other research projects on alcoholism in the Mexican-American population [30] and pharmacogenomics of CYP enzymes in both Mexican-Americans and African-Americans [31,32]. Genotypes of the 10 selected markers on the 90 Mexican-American and 90 African-American subjects were determined by using the thin-film biosensor chip platform. Representative images of the different genotypes from different individuals are shown in Figure 3E, 3F, and 3G. Genotypes of each individual on the 10 markers were saved in linkage format and uploaded to HaploView. Observed genotype frequencies, allele frequencies, expected heterozygosity, and Hardy-Weinberg p-value of the 10 markers is summarized in Table 5. No significant HW p-values (<0.0010) were found. No TT deletion mutation at 1302 and C mutation at rs8192874 were detected in these two population samples. This indicates that these mutations have very low frequencies in the general populations. The genotyping data of the 8 htSNPs were uploaded to HaploView 3.12 to define LD patterns and haplotypes structures of CYP7A1. In Mexican-Americans, three LD blocks were identified. In comparison to the CEU LD blocks, MA-B3 in the distal promoter region has the same pattern as CEU-B2, but haplotype frequencies are different. MA-B3H1 has a frequency (78%) higher than CEU-B2H1 (55%). Unlike one big block in CEU, the CYP7A1 gene is divided by two LD blocks in the Mexican-American population. MA-B2 covers from proximal promoter to intron 2, whereas MA-B1 extents from 3'-UTR to 3'-downstream. The recombination frequencies between the blocks are 80–90%. In African-Americans, two LD blocks were recognized. AA-B2 has the identical structure as YRI-B2 and frequencies of the two haplotypes (AA-B2H1 and AA-B2H1) in the African-American population are almost the same as YRI-B2H1 and YRI-B2H2. AA-B1 is shorter than YRI-B1. The HapMap htSNPs are necessary SNP markers to capture all haplotypes in the MA and AA populations.

In summary, the human CYP7A1 gene and its surrounding sequences have constant genetic structures across all populations. This genetic structure can be divided into three components: (1) the distal promoter region, about 7-kb upstream from the transcriptional start code, there is a 3-kb LD block highly conserved across all populations. Only two haplotypes exist in this region in the most populations, except YRI. The most common haplotype in CEU and Mexican-American becomes the second most common haplotypes in YRI, African-American, CHB, and JPT populations. (2) A relative conserved LD block is present in the proximal part of CYP7A1 gene from the proximal promoter region (about 500 bp from the transcriptional start code) to intron 2 of CYP7A1. The two most conserved haplotypes count for up to 80 to 90% of the haplotype frequencies in all populations in this region. (3) A much diverted LD pattern is observed in the lower part of the CYP7A1 gene (from intron 4 to 3'-downstream). In CEU and YRI, a complete or partial LD block is merged to the block in the proximal part of the CYP7A1 gene. In Mexican-Americans, a LD block in this region is separated from the block in the proximal part of the CYP7A1 gene. In JPT, a weak linkage makes the proximal part block extended into the 3'-UTR. In CHB and African-Americans, there is no LD existing in this area.

## Conclusion

Here we demonstrate a genetic approach to analyze LD patterns and haplotype blocks in CYP7A1 gene. Various degree of LD is found across different regions in different populations. A set of htSNPs is identified that can be used in an association study to capture common haplotypes in different populations. An inexpensive genotyping platform on thin-film biosensor chips is established to genotype the htSNPs. This chip technology can be applied in any laboratory with basic molecular genetic setting. The defined haplotype block structure in CYP7A1 gene may lead to a better design for genetic association studies to correlate genetic variations in CYP7A1 gene to cholesterol and bile acid metabolism and human diseases, such as gallstone disease. Because of high polymorphism and strong LD in the promoter region of CYP7A1, it should be considered in future studies to evaluate which CYP7A1 promoter haplotypes are more efficient for transcriptional regulation by its regulatory factors, such as FXR, LXR, RXR, PXR, SHP, and LRH1.

## Methods

### Human subject

The DNA samples for the HapMap come from a total of 270 people: 90 individuals from the Yoruba of Ibadan, Nigeria (YRI), (30 sets of trios, each trio with samples from two parents and an adult child); 90 individuals (30 sets of trios) from U.S. residents with northern and western European ancestry collected by the Centre d'Etude du Polymorphisme Humain (CEU); 45 unrelated individuals from the Tokyo area in Japan (JPT); and 45 unrelated individuals from Beijing, China (CHB). Two American population samples of Mexican-Americans and African-Americans were also used in this study. These DNA samples were collected by other research projects on alcoholism in the Mexican-American population [30] and pharmacogenomics of CYP enzymes in African-Americans [32]. Studies on these human subjects were approved by the Human Subjects Committee of the Kansas University Medical Center. Ninety DNA samples of healthy individuals were randomly selected from each population.

### Analysis of linkage disequilibrium blocks and haplotype structure

LD patterns and haplotype structures of CYP7A1 gene in the HapMap populations were analyzed by using genotype data from the HapMap database. In the HapMap Phase II database, a total of about 5.9 million SNPs (about 1 SNP every 500 bp across the genome) are typed in the four HapMap populations [21]. Genotypes of each selected SNP in the 270 HapMap population samples can be downloaded from its database [34]. Fourteen SNPs are genotyped by the HapMap project in a total of 25 kb region with 10 kb of 5'-upstream flanking, 5 kb of 3'-downstream flanking, and the CYP7A1 gene sequences. The SNP density is about 1.8 kb per SNP. The CYP7A1 A-278C (or A-204C) promoter polymorphism is included with an ID number of rs3808607. A close promoter polymorphism C-554T, which was identified together with A-278C by Wang et al. [7], is also included as rs3824260. Chromosomal positions and locations in the CYP7A1 gene regions of the 14 SNPs are listed in Table 1 with polymorphic allele 1 for common allele and allele 2 for less common allele in CEU. Genotypes of each individual samples for the 14 SNP markers were dumped from the HapMap database and saved as a HapMap formatted file that can be opened directly by HaploView 3.12 for defining LD patterns and haplotype structure [25]. Four HapMap files were separately saved for CEU, CHB, JPT, and YRI, respectively. By uploading the files into HaploView, frequencies of allele 2, frequencies of observed heterozygous genotypes, and Hardy-Weinberg *p*-value for each marker were summarized for each population (see Table 2). No marker has a HW *p*-value smaller than the cutoff value of 0.0010 in the four populations. The LD between any two markers was defined by HaploView 3.12. A standard color scheme is used to display LD in Figure 1. A LD block was created by confidence intervals [25] if 95% of the informative comparisons are in strong LD using default algorithms of 95% confidence bounds on D prime. Haplotypes structure was defined by using an accelerated EM algorithm, similar to the partition/ligation method [33]. This creates highly accurate population frequency estimates of the phased haplotypes, based on the maximum likelihood as determined from the unphased input. Haplotypes with frequency > 2% in a block in CEU, YRI, JPT, and CHB are displayed in Figure 2. Alleles with blue boxes and red boxes represent common alleles and less common alleles in CEU, defined as Allele 1 and Allele 2, respectively. In the crossing areas, a value of multiallelic D' is shown to represent the level of recombination between the two blocks.

**Table 1:** Chromosomal positions and gene locations of the 14 CYP7A1 SNPs

#	SNP ID	Chromosome position	Location in CYP7A1 gene	Variation* Allele 1/2
1	rs10504255	59448422	3'-downstream	A/G
2	rs10957057	59450301	3'-downstream	C/T
3	rs8192879	59453537	3'-UTR	C/T
4	rs11786580	59455901	Intron 4	C/T
5	rs3747809	59456945	Intron 4	T/C
6	rs1457043	59460400	Intron 2	T/C
7	rs2162459	59461003	Intron 1	A/G
8	rs3808607	59462885	5'-upstream	T/G
9	rs3824260	59463151	5'-upstream	G/A
10	rs7833904	59467623	5'-upstream	T/A
11	rs3903445	59468303	5'-upstream	G/T
12	rs1125226	59469472	5'-upstream	A/C
13	rs1023649	59470379	5'-upstream	A/G
14	rs1023652	59470577	5'-upstream	C/G

* Variation allele sequences are based on the plus strand sequence.

**Table 2:** Genotype frequencies of the 14 CYP7A1 SNPs in the four HapMap populations.

#	SNP ID	Variation Allele 1/2	CEU			CHB			JPT			YRI		
			FA2	ObH	HW-p	FA2	ObH	HW-p	FA2	ObH	HW-p	FA2	ObH	HW-p
1	rs10504255	A/G	0.317	0.456	1.0	0.200	0.356	0.886	0.170	0.250	0.694	0.017	0.022	1.0
2	rs10957057	C/T	0.142	0.222	1.0	0.122	0.244	0.999	0.159	0.318	0.586	0.092	0.178	1.0
3	rs8192879	C/T	0.367	0.467	0.754	0.267	0.444	0.663	0.261	0.386	1.0	0.225	0.333	1.0
4	rs11786580	C/T	0.242	0.367	1.0	0.056	0.111	1.0	0.091	0.182	1.0	0.192	0.311	1.0
5	rs3747809	A/G	0	0		0.011	0.022	1.0	0.058	0.116	1.0	0	0	
6	rs1457043	T/C	0.392	0.522	0.754	0.567	0.556	0.616	0.625	0.523	0.718	0.575	0.467	0.911
7	rs2162459	A/G	0.392	0.522	0.754	0.567	0.556	0.616	0.625	0.523	0.718	0.583	0.456	1.0
8	rs3808607	T/G	0.367	0.489	1.0	0.411	0.467	0.989	0.570	0.535	0.836	0.583	0.456	1.0
9	rs3824260	G/A	0.367	0.489	1.0	0.411	0.467	0.989	0.570	0.535	0.836	0.550	0.478	0.784
10	rs7833904	T/A	0.408	0.511	1.0	0.622	0.533	0.612	0.663	0.535	0.382	0.558	0.444	0.313
11	rs3903445	G/T	0.408	0.511	1.0	0.622	0.533	0.612	0.648	0.523	0.580	0.550	0.467	0.44
12	rs1125226	A/C	0.398	0.517	1.0	0.622	0.533	0.612	0.663	0.535	0.382	0.575	0.456	0.343
13	rs1023649	A/G	0.415	0.517	1.0	0.622	0.533	0.612	0.648	0.523	0.580	0.575	0.456	0.343
14	rs1023652	C/G	0.408	0.511	1.0	0.433	0.556	0.616	0.314	0.488	0.665	0.125	0.189	0.073

FA2: frequency of allele 2; ObH: observed frequency of heterozygous genotypes; HW-p: Hardy-Weinberg p value.

### Genotyping on thin-film biosensor chip

For each selected SNP, target DNA molecules from each sample were amplified by PCR. PCR primers were designed based on the following criteria to make the PCR reaction uniform: (1) product size should be 120–200 bp with about 50–100 base flanking sequences around the SNP site in both directions, and (2) annealing temperature should be about 60°C for a standard PCR reaction condition. The best primer sets were selected by DS Gene Software version 1.5 (accelrys). The primer sequences for each SNP site are listed in Table 4. The selected primer sequences were synthesized by Invitrogen (Carlsbad, California). Multiple sets of the PCR products were amplified in a single PCR reaction.

For each SNP, three oligonucleotide probes were synthesized. A pair of allele specific P-1 oligos, differing only in their 3'-terminal nucleotide sequence, generally has 40 nucleotides complementary to the corresponding target sequences, and an additional 10-dA residue at their 5'-ends that constitutes a "spacer". Their 5'-terminal nucleotide is modified with an aldehyde group, allowing covalent attachment to the chip surface [27]. A second oligonucleotide probe (biotin-P2) with 20 nucleotides immediately adjacent to the SNP nucleotide carries a biotin at the 3' end for detection, and a phosphate at its 5' end for ligation. To test genotyping specificity, a pair of oligonucleotide targets was also synthesized. The P1, P2 and target sequences for each SNP are listed in Table 4. The synthesized P1 oligos were dissolved to 100 μM in 0.1 M phosphate buffer, pH 7.8. A P1 working solution of 1 μM in 0.1 M phosphate buffer, pH 7.8, and 10% glycerol was prepared for each P-1 probe before spotting. Twenty nano liter of the P1 working solution was spotted on a 7 × 7 mm^2 ^chip in an 8 per row × 6 per column format, by a BioDot PC controlled dispense arrayer AD3200. A duplicate set of P1 probes were spotted on a chip with a spotting pattern shown in Figure 3A. After the spotted chips were incubated in a humidity-controlled chamber for at least 2 hrs, the chips were washed with 0.1% SDS, water, and air dried. A standard operating procedure for genotyping SNPs on the printed biosensor chips was described previously [27]. An arrayed chip was assembled into a square well of a 96-well microtiter plate for hybridization. A ligation reaction was carried out in a microtiter plate well containing an arrayed chip. A reaction solution (100 μl) contained 100 femtomoles of each relevant PCR amplicon of the 10 CYP7A1 SNPs, 10 nM P-2 probe (one for each SNP) and 5 units of mutant Ampligase in a buffer of 20 mM Tris-HCl, pH 8.3, 25 mM KCl, 10 mM MgCl_2_, 0.5 mM NAD, 0.01% Triton X-100, and 5 mg/ml alkaline treated casein. The ligation reaction was incubated for 20 min at 60°C. 96 chips in a 96-well plate were processed simultaneously. After a stringent wash (3 times in 0.01 M NaOH at room temperature and 3 times in 0.1 × SSC), the chips were incubated with an antibiotin-horse radish peroxidase (HRP) conjugate (1 μg/ml in hybridization buffer) for 10 min, and the chips were rinsed with 0.1 × SSC. 100 μl of a precipitate-generating HRP substrate TMB (BioFx) was added to each chip and incubated for 5 min, rinsed in ddH_2_0, and air-dried.

## Authors' contributions

KN carried out the genotyping experiments. SW provided assistance on haplotype analysis. RDJ provide thin-film biosensor chip for genotyping and participated in the experiment design. GLG participated in the design of the study and data analysis. YYW provided Mexican-American and African-American samples for this study. CDK participated in study design and help preparation of manuscript. XBZ coordinated the experimental design and was responsible for quality control, data analysis and manuscript preparation. All authors read and approved the final manuscript.

**Figure 4:**
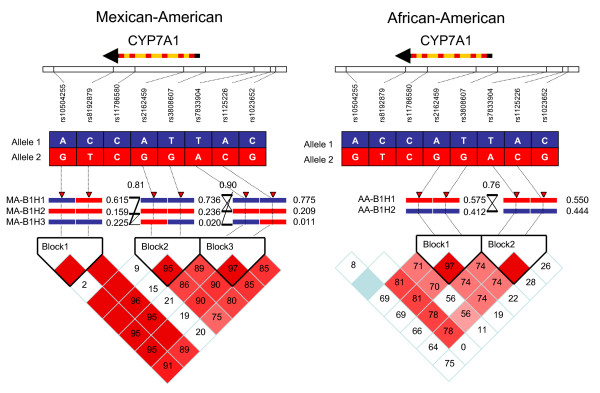
LD patterns and haplotype structures of the CYP7A1 gene in Mexican-American and African-American populations.

**Table 3:** Ten selected genetic variations for CYP7A1 genotyping.

SNP	Allele 1/2	Position	Note
rs10504255	A/G	3'-downstream	HapMap htSNP in CEU
rs8192879	C/T	3'-UTR	HapMap htSNP in CEU, JPT, and YRI
1302 delTT	TT/--	Exon 6	Frameshift, CYP7A1 deficiency, high hepatic cholesterol content
rs11786580	C/T	Intron 4	HapMap htSNP in CEU, JPT, and YRI
rs8192874	C/T	Exon 3	Non-synonymous SNP, Asn33Ser
rs216245953	A/G	Intron 1	HapMap htSNP in JPT
rs3808607	T/G	Promoter	HapMap htSNP in CEU and JPT
rs7833904	T/A	5-upstream	HapMap htSNP in YRI
rs1125226	A/C	5-upstream	HapMap htSNP in JPT
rs1023652	C/G	5'-upstream	HapMap htSNP in CEU

**Table 4:** Oligonucleotide sequences of capture probe P1, detection probe P2, synthetic targets, and PCR primers.

SNP	Oligonucleotide sequence
rs10504255 P1-A	ALD-AAAAAAAAAAAACCTGAGCACTAGCCAGCTGTGTTCTCAATTCTGTGTGTAA
rs10504255 P1-G	ALD-AAAAAAAAAAAACCTGAGCACTAGCCAGCTGTGTTCTCAATTCTGTGTGTAG
rs10504255 P2	Phosphate-CTCTTTCCCAATATTTCAAT-biotin
rs10504255 Target for P1-A	ATTGAAATATTGGGAAAGAGTTACACACAGAATTGAGAACA
rs10504255 Target for P1-G	ATTGAAATATTGGGAAAGAGCTACACACAGAATTGAGAACA
rs10504255 Forward	TAAACCTGCCTTGTCACGC
rs10504255 Reverse	AGTTGCAAACGCTGGTTG
	
rs8192879 P1-C	ALD-AAAAAAAAAAGAAAAAACAATCTGCCAATTAGAATACATATCTTTTCTTC
rs8192879 P1-T	ALD-AAAAAAAAAAGAAAAAACAATCTGCCAATTAGAATACATATCTTTTCTTT
rs8192879 P2	Phosphate-GGAGACGGGATCTCACTAAG-biotin
rs8192879 Target for P1-C	CTTAGTGAGATCCCGTCTCCGAAGAAAAGATATGTATTCTA
rs8192879 Target for P1-T	CTTAGTGAGATCCCGTCTCCAAAGAAAAGATATGTATTCTA
rs8192879 Forward	ACCTGTAGTCTTAGCTACTCG
rs8192879 Reverse	CCTTAGGAAAAAACAATCTGCC
	
rs11786580 P1-C	ALD-AAAAAAAAAACATTTACAAGAACCTATTTTTATCCATATACTATCTGATGC
rs11786580 P1-T	ALD-AAAAAAAAAACATTTACAAGAACCTATTTTTATCCATATACTATCTGATGT
rs11786580 P2	Phosphate-TGCGTGGCGATCACCGCTGC-biotin
rs11786580 Target for P1-C	GCAGCGGTGATCGCCACGCAGCATCAGATAGTATATGGATA
rs11786580 Target for P1-T	GCAGCGGTGATCGCCACGCAACATCAGATAGTATATGGATA
rs11786580 Forward	TCTAGGTGTTTAATGCAGCTTC
rs11786580 Reverse	GTCCCTTTATGCCTTTACCG
	
rs8192874 P1-C	ALD-AAAAAAAAAATTCTCGTGCCTCAAGCTCTCTGCCAGTTTCTCCCGGGCAC
rs8192874 P1-T	ALD-AAAAAAAAAATTCTCGTGCCTCAAGCTCTCTGCCAGTTTCTCCCGGGCAT
rs8192874 P2	Phosphate-TGTGCGCAGTCCTGAACATG-biotin
rs8192874 Target for P1-C	CATGTTCAGGACTGCGCACAGTGCCCGGGAGAAACTGGCAG
rs8192874 Target for P1-T	CATGTTCAGGACTGCGCACAATGCCCGGGAGAAACTGGCAG
rs8192874 Forward	TCAGTTCTGAGATGCTTTCCC
rs8192874 Reverse	AGTCTTTCCAGCCCTGGTAG
	
rs2162459 P1-A	ALD-AAAAAAAAAATCTCTAGAGGTGGTTCACCCGTTTGCCTGTCAGACACAAA
rs2162459 P1-G	ALD-AAAAAAAAAATCTCTAGAGGTGGTTCACCCGTTTGCCTGTCAGACACAAG
rs2162459 P2	Phosphate-TGTATGATAGACATGGATGA-biotin
rs2162459 Target for P1-A	TCATCCATGTCTATCATACATTTGTGTCTGACAGGCAAACG
rs2162459 Target for P1-G	TCATCCATGTCTATCATACACTTGTGTCTGACAGGCAAACG
rs2162459 Forward	AAATTGCAGAGCACAGCC
rs2162459 Reverse	TCAAAGTTTGAAGTCAGTGGG
	
rs3808607 P1-G	ALD-AAAAAAAAAACAAAGCAATCAGAGACCTGCAATACTTGATAAGTTGAAGG
rs3808607 P1-T	ALD-AAAAAAAAAACAAAGCAATCAGAGACCTGCAATACTTGATAAGTTGAAGT
rs3808607 P2	Phosphate-TCTCTCAAATATATGTTGAC-biotin
rs3808607 Target for P1-G	GTCAACATATATTTGAGAGACCTTCAACTTATCAAGTATTG
rs3808607 Target for P1-T	GTCAACATATATTTGAGAGAACTTCAACTTATCAAGTATTG
rs3808607 Forward	AGTCCACAGGTATCAGAAGTG
rs3808607 Reverse	CCCCAGGTCCGAATGTTAAG
	
rs7833904 P1-A	ALD-AAAAAAAAAACAGGAGAAAGCGTAAGTGCCCTGACAAAGAAAAGAGGTAA
rs7833904 P1-T	ALD-AAAAAAAAAACAGGAGAAAGCGTAAGTGCCCTGACAAAGAAAAGAGGTAT
rs7833904 P2	Phosphate-AGTTGCCAGAAAACCTGGCA-biotin
rs7833904 Target for P1-A	TGCCAGGTTTTCTGGCAACTTTACCTCTTTTCTTTGTCAGG
rs7833904 Target for P1-T	TGCCAGGTTTTCTGGCAACTATACCTCTTTTCTTTGTCAGG
rs7833904 Forward	AATGTGAGCAAGAAAGCCC
rs7833904 Reverse	GAACTCAGTTTATTTTGCCAGG
	
rs1125226 P1-A	ALD-AAAAAAAAAACAACTGGTTCATCAGACTTGCTGCGACCAATTAACCTTGA
rs1125226 P1-C	ALD-AAAAAAAAAACAACTGGTTCATCAGACTTGCTGCGACCAATTAACCTTGC
rs1125226 P2	Phosphate-CAGCTCAGGGAGAGAGAGAG-biotin
rs1125226 Target for P1-A	CTCTCTCTCTCCCTGAGCTGTCAAGGTTAATTGGTCGCAGC
rs1125226 Target for P1-C	CTCTCTCTCTCCCTGAGCTGGCAAGGTTAATTGGTCGCAGC
rs1125226 Forward	TTTCTCTCTCTCCCTCTTTCTC
rs1125226 Reverse	CTGGTTCATCAGACTTGCTG
	
rs1023652 P1-C	ALD-AAAAAAAAAAAAAATTCAAGTACCATAATATTCACCCCTTTAAAGAGTAC
rs1023652 P1-G	ALD-AAAAAAAAAAAAAATTCAAGTACCATAATATTCACCCCTTTAAAGAGTAG
rs1023652 P2	Phosphate-AATTCAAAATTTCTAGCATA-biotin
rs1023652 Target for P1-C	TATGCTAGAAATTTTGAATTGTACTCTTTAAAGGGGTGAAT
rs1023652 Target for P1-G	TATGCTAGAAATTTTGAATTCTACTCTTTAAAGGGGTGAAT
rs1023652 Forward	TGGAATGCCAGTTACCCCAC
rs1023652 Reverse	GGTGATGGTCACACAATCTTTG
	
1302 delTT P1-TT	ALD-AAAAAAAAAATGTATTTTTATTGCAGACTTTTAAATATGATAGGTATCTT
1302 delTT P1-DEL	ALD-AAAAAAAAAATTTGTATTTTTATTGCAGACTTTTAAATATGATAGGTATC
1302 delTT P2	Phosphate-GATGAAAACGGGAAGACAAA-biotin
1302 delTT Target for P1-TT	TGTCTTCCCGTTTTCATCAAGATACCTATCATATTTAAAA
1302 delTT Target for P1-DEL	TGTCTTCCCGTTTTCATCGATACCTATCATATTTAAAA
1302 delTT Forward	ACTAGCTTAAAGGCGGTTTTC
1302 delTT Reverse	CGATCCAAAGGGCATGTAG

* Shadows indicate SNP nucleotides in P1 probe and target sequences.

**Table 5:** Allele frequencies of the selected CYP7A1 htSNPs and mutation markers A. in the Mexican-American population.

#	SNP ID	Variation Allele 1/2	Observed genotype frequencies			Observed allele frequencies		Hardy-Wenberg	
			
			Homo Allele 1	Hetero	Homo Allele 2	Allele 1	Allele 2	Expect heterozygous	P value
1	rs10504255	A/G	0.71	0.25	0.03	0.84	0.16	0.27	0.79
2	rs8192879	C/T	0.41	0.42	0.18	0.61	0.39	0.47	0.34
3	rs11786580	C/T	0.75	0.25	0	0.87	0.13	0.22	0.45
4	rs8192874	T/C	1.00	0	0	1.00	0		
5	rs2162459	A/G	0.59	0.31	0.10	0.75	0.25	0.38	0.13
6	rs3808607	T/G	0.62	0.30	0.09	0.76	0.24	0.37	0.09
7	rs7833904	T/A	0.64	0.30	0.07	0.79	0.21	0.33	0.37
8	rs1125226	A/C	0.62	0.30	0.08	0.78	0.22	0.34	0.18
9	rs1023652	C/G	0.67	0.26	0.07	0.81	0.19	0.32	0.14
10	1302 delTT	TT/–	1.00	0	0	1.00	0		

B. in the African-American population									

1	rs10504255	A/G	0.81	0.18	0.01	0.84	0.10	0.18	1.00
2	rs8192879	C/T	0.56	0.36	0.08	0.61	0.26	0.38	0.82
3	rs11786580	C/T	0.60	0.36	0.04	0.87	0.22	0.34	0.90
4	rs8192874	T/C	1.00	0	0	1.00	0		
5	rs2162459	A/G	0.25	0.34	0.41	0.75	0.58	0.49	0.01
6	rs3808607	T/G	0.23	0.36	0.40	0.76	0.58	0.49	0.03
7	rs7833904	T/A	0.20	0.50	0.30	0.79	0.55	0.50	1.00
8	rs1125226	A/C	0.20	0.49	0.31	0.78	0.56	0.49	1.00
9	rs1023652	C/G	0.33	0.38	0.29	0.81	0.49	0.50	0.04
10	1302 delTT	TT/--	1.00	0	0	1.00	0		

* Shadows indicate SNP nucleotides in P1 probe and target sequences.
